# Chemomechanical Origin of Morphological Disparity in Lithium Metal Electrodeposition

**DOI:** 10.1002/adma.202522026

**Published:** 2026-02-15

**Authors:** Yaobin Xu, Ruyue Fang, Dingchuan Xue, Hao Jia, Phung M. L. Le, Ji‐Guang Zhang, Wu Xu, Sulin Zhang, Chongmin Wang

**Affiliations:** ^1^ Energy and Environment Directorate Pacific Northwest National Laboratory Richland Washington USA; ^2^ Department of Engineering Science and Mechanics Pennsylvania State University University Park Pennsylvania USA; ^3^ Environmental Molecular Sciences Laboratory Pacific Northwest National Laboratory Richland Washington USA

**Keywords:** cryo‐TEM, Li morphology, phase field simulation, solid electrolyte interphase

## Abstract

The morphology of electrochemically deposited lithium (Li) critically governs the cycling stability and safety of Li metal batteries, yet the underlying controlling factors remain poorly understood. Even within the same coin cell, Li deposits can exhibit strikingly different morphologies, for example, sparse whiskers coexisting with particle‐like deposits, indicting strong local variations in growth conditions. By combining cryogenic transmission electron microscopy with phase‐field modeling, here we identify the root cause of this disparity. We reveal that the morphological divergence originates from the variations in structure and chemical composition of the initial SEI layer formed on the copper (Cu) substrate. Solvent‐derived organic SEI favors whisker growth, whereas salt‐derived SEI promotes particle formation. This study establishes a direct mechanistic link between SEI chemomechanical properties and Li morphology, providing design principles for tailoring SEI layers to control Li deposition.

## Graphical abstract

Selective dissociation of either solvent or salt on copper current collector induced different solid electrolyte interphase (SEI) layers. The solvent derived organic SEI favors formation of Li whisker, while salt derived SEI favors formation of Li particle, revealing the root cause of Li morphology disparity within the same cell.

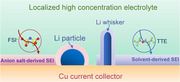

## 1 Introduction

The growing demands in portable electronics, electric vehicles, and smart grids, together with the global push for renewable energy, are driving the development of advanced energy storage systems requiring higher energy density, higher power density, and longer cycling life [1, 2, 3, 4]. Lithium (Li) metal is a leading candidate anode for such high‐energy batteries due to its extremely low density (0.534 gcm^−3^), exceptionally negative electrochemical potential (−3.040 V versus standard hydrogen electrode), and ultrahigh theoretical specific capacity (3860 mAhg^−1^), significantly exceeding that of commercial graphite anode (372 mAhg^−1^). Consequently, Li metal anode is essential for next‐generation technologies such as Li||Li transition metal oxides, Li||S and Li||air batteries [5, 6, 7, 8].

However, it has been shown that Li deposits exhibit diverse morphologies, including mossy, particulate, whisker‐like, and dendrite structures that critically impact battery performance and safety. Dendrites, characterized by sharp protrusions, pose a major safety hazard by penetrating the separator and causing internal short circuits, while the morphology‐dependent surface area governs the extent of detrimental side reactions between Li and electrolytes, depleting active Li and electrolytes and accelerating capacity fade [5, 7, 9]. Therefore, controlling Li deposition morphology is paramount for improving cycle life, though the fundamental factors dictating it remain poorly understood [10, 11, 12, 13, 14, 15].

During Li deposition, a thin solid electrolyte interphase (SEI) layer forms on the surface of deposition substrates (e.g., Li metal anode and Cu current collector, termed SEI_Li_ and SEI_Cu_, respectively) whenever Li or Cu contacts the electrolytes, due to electrochemical decomposition [6, 16, 17]. Li^+^ ions first migrate through the SEI and are then reduced to metallic Li, depositing between the SEI layer and the Li metal anode or Cu. The SEI layer blocks electron conduction while allowing Li^+^ ion transport, thereby preventing continuous reactions between the electrolyte and the freshly formed Li metal beneath the SEI. In this way, the SEI layer regulates both Li^+^ ion distribution from the electrolyte and electron distribution at the anode surface. The properties and stability of the SEI layer are tightly coupled to the local ionic concentration field and electric field at the reaction interfaces, significantly affecting Li metal nucleation and growth kinetics, and ultimately dictating Li morphology [6, 11, 18]. While it is clear that the SEI layer plays a decisive role in shaping Li deposits, the detailed relationship between SEI characteristics and Li morphology remains poorly understood.

Here we explore how the chemomechanical properties of the SEI layer modulate Li deposition morphology. By using battery cells with the same electrode pairs but different electrolytes, we generate SEI layers with varied chemical compositions and mechanical properties, which in turn produce distinct Li morphologies. In particular, within the same coin cell, we observed the coexistence of Li particles and Li whiskers. Detailed analyses of the SEI on these different Li nanostructures reveal key factors governing morphological evolution. Phase field simulations further establish a strong correlation between chemomechanics and Li morphology. Our findings offer a promising pathway to tailor Li deposit morphology by tuning SEI chemomechanics, guiding the rational design of electrolytes for high‐performance Li metal batteries.

## 2 Results and Discussion

### 2.1 Tuning Li Morphology through Electrolyte Chemistry

To generate distinct Li deposit morphologies, we designed battery cells with two different electrolytes. The first electrolyte, referred to as a localized high concentration electrolyte (LHCE), consists of lithium bis(fluorosulfonyl)imide (LiFSI) salt, 1,2‐dimethoxyethane (DME) solvent and 1,1,2,2‐tetrafluoroethyl‐2,2,3,3‐tetrafluoropropyl ether (TTE) as the diluent, in a molar ratio of 1:1.2:3. The second electrolyte, designated as carbonate‐based low concentration electrolyte (LCE), is composed of 1.2 M LiPF_6_ in ethylene carbonate (EC)/ethyl methyl carbonate (EMC) (3:7 by wt.) with 5 wt.% vinylene carbonate (VC). Copper (Cu) transmission electron microscopy (TEM) grids were used as the counter electrode of Li metal, and Li was electrochemically deposited on Cu TEM grids at a current density of 0.1 mA cm^−2^ for 100 min.

**FIGURE 1:**
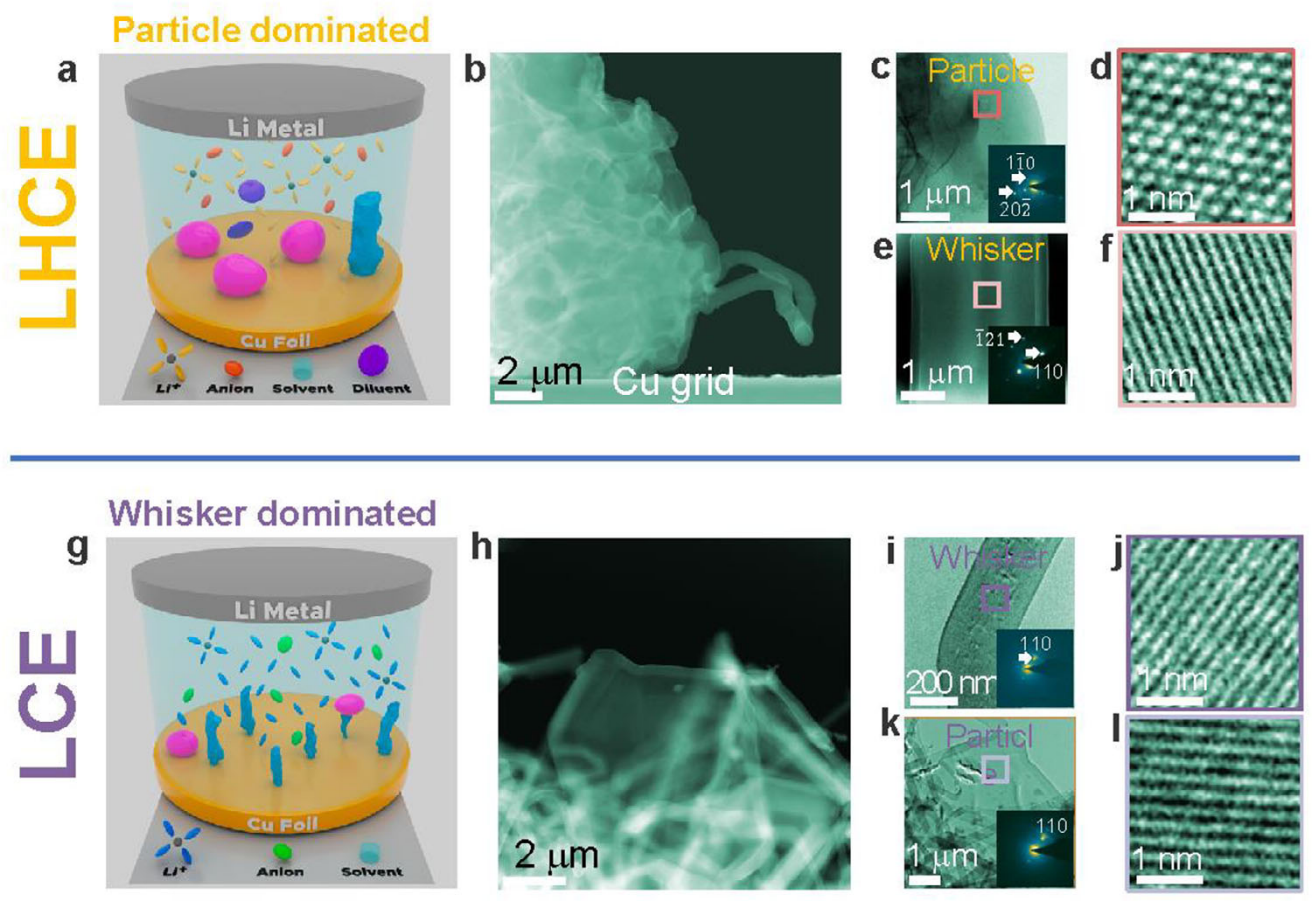
Morphology of Li deposits in LHCE and LCE. (a) Schematic drawing shows the Li morphology is dominated by particle with small fraction of whisker in the ether‐based LHCE. (b) Cryo‐STEM image of Li deposits; (c) Bright‐field TEM image of Li particle. (d) High‐resolution TEM image of Li particle; (e) Bright‐field TEM image of Li whisker, (f) High‐resolution TEM image of Li whisker. (g) Schematic drawing shows the Li morphology is dominated by whisker with small fraction of particle in the low concentration carbonate‐based electrolyte. (h) Cryo‐STEM image of Li deposits; (i) Bright‐field TEM image of Li whisker. (j) High‐resolution TEM image of Li whisker; (k) Bright‐field TEM image of Li particle, (l) High‐resolution TEM image of Li particle.

As shown in Figure 1, the Li deposits formed in the LCE and LHCE are crystalline Li metal (Figure 1c–f,i–l). Morphologically, in the LHCE, Li deposits predominantly form as particles, with very few Li whiskers (Figure 1a,b; Figure S1a) [19]. In contrast, in the LCE, Li deposits mainly appear as whiskers, with a minor fraction of Li particles (Figure 1g,h; Figure S1b) [20, 21]. The co‐existence of Li whiskers and Li particles within the same electrolyte environment provides a unique opportunity to directly correlate SEI properties with Li morphology (Figure 1). In the following sections, we analyze the SEI characteristics on Li particles and Li whiskers formed in both LHCE and LCE.

### 2.2 Microstructure and Composition of SEI Layer on Li Particles and Li Whiskers

**FIGURE 2:**
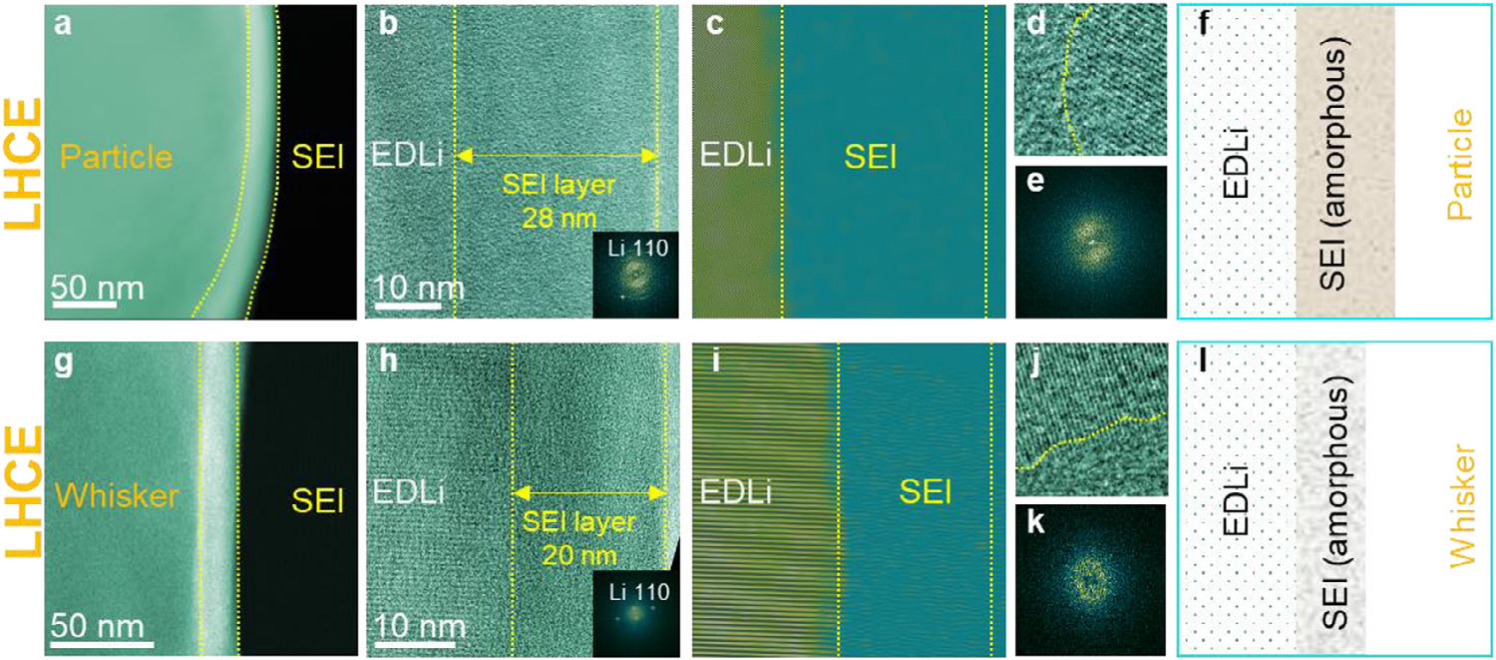
Structure of SEIs formed on Li particle and whisker formed in the ether‐based LHCE. (a) STEM image of Li whisker shows the SEI layer. (b) TEM image shows the SEI layer on Li particle, the inset is FFT. (c) Filtered TEM image shows the SEI layer. (d) HRTEM image showing interface between SEI layer the Li. (e) FFT of c. (f) schematic drawing shows the Li whisker with SEI layer. (g) STEM image of Li particle shows the SEI layer. (h) TEM image shows the SEI layer on Li particle, the inset is FFT. (i) Filtered TEM image shows the SEI layer. (j) HRTEM image shows interface between Li and SEI layer. (k) FFT of i. (l) schematic drawing shows the Li particle with SEI layer.

In the ether‐based LHCE, Li particles are the dominant morphology. The SEI layer on these Li particles, approximately 28 nm thick (Figure 2a,b) is primarily derived from salt anions, and fully amorphous (Figure 2c–f). The monolithic structure (Figure 2c–f) differs from previously proposed mosaic and multilayer models [22, 23]. Compositional analyses using energy dispersive X‐ray spectroscopy (EDS) and electron energy loss spectroscopy (EELS) (Figures S2a,b and S3, Figure S4a,b and Table S1) show that the SEI on these Li particles is rich of S and Li, but low in C, indicating that is mainly originates from FSI^−^ salt anions rather than solvent. Elemental mapping by EELS (Figure S4) shows that sulfur (S) and Li are concentrated in the outer SEI layer, while oxygen (O) is concentrated in the inner layer, suggesting the possible presence of Li_2_S, SO_2_F, and SO_x_ species. It is noteworthy that both sulfur (S) and fluorine (F) originate from the decomposition of FSI^−^ anion. Consequently, one might expect that the atomic ratio of S to F in SEI to be approximately 1:1. However, our measurements consistently show that the concentration of S is higher than that of F (Figures S2 and S3). This discrepancy may be attributed to two factors. First, S and F may form distinct compounds in the SEI layer, resulting in different retention or trapping efficiencies for their respective species. Second, S and F likely exhibit different spatial distributions within the SEI, indicating a localized concentration effect. These observations highlight the need for further investigation into the chemical bonding environment and distribution profiles of S‐ and F‐containing species, even in systems with predominantly amorphous SEI structures.

In contrast, the SEI layer on Li whiskers formed in LHCE is primarily solvent‐derived (Figure 2g–l). The SEI exhibits monolithic amorphous structure of ∼20 nm thick (Figure 2g–l). EDS and EELS analyses (Figure S2c,d and S3, Figure S4c,d, and Table S1) show a higher C content, confirming solvent‐derived SEI.

**FIGURE 3:**
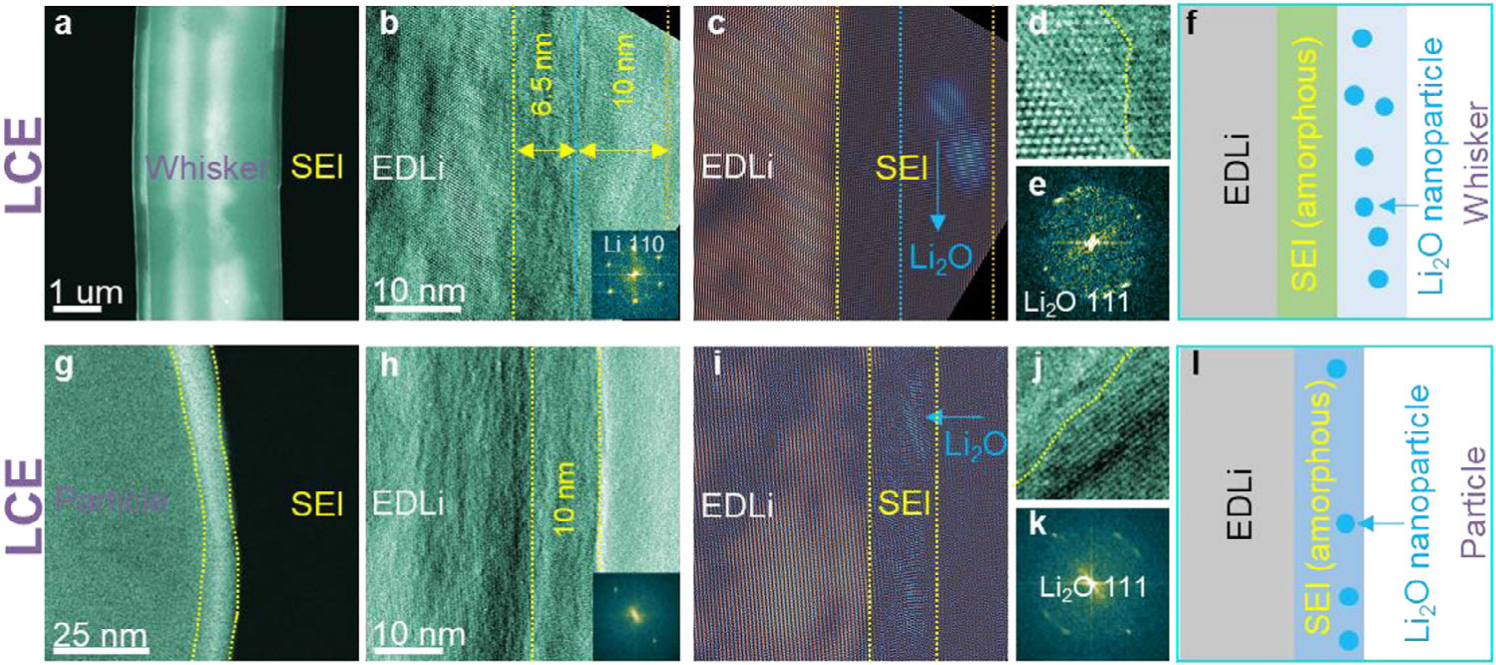
Structure of SEIs formed on Li whisker and particle formed in the carbonate‐based LCE. (a). STEM image of Li particle shows the SEI layer. (b) TEM image shows the SEI layer on Li particle, the inset is FFT. (c) Filtered TEM image shows the SEI layer and Li_2_O dispersed in the SEI layer. (d) HRTEM image shows the interface between Li particle and SEI layer. (e) FFT of c. (f) schematic drawing shows the Li particle with SEI layer. (g) STEM image of Li whisker shows the SEI layer. (h) TEM image shows the SEI layer on Li whisker, the inset is FFT. (i) Filtered TEM image shows the SEI layer and Li_2_O dispersed in the top SEI layer. (j) HRTEM image shows the interface between Li and SEI layer. (k) FFT of i. (l) schematic drawing shows the Li whisker with SEI layer.

In the carbonate‐based LCE, Li whiskers are the dominant morphology, with a small fraction of Li particles (Figure 1b; Figure S1b). Similar as LHCE, the SEI on Li particles is primarily derived from salt anions, while that on Li whiskers from solvent (Figures S5–S7 and Table S2). Structurally, the SEI layer on Li whiskers in LCE is ∼16.5 nm thick (Figure 3a–f) with an amorphous inner layer of ∼6.5 nm thick and a mosaic outer layer of ∼10 nm containing dispersed Li_2_O particles (Figure 3c–f). EDS analysis confirms high concentration of C, indicating a solvent‐derived SEI (Figure S6b). The SEI layer on Li particles in LCE exhibits mosaic structure (Figure 3g–l), featuring dispersed Li_2_O nanoparticles embedded in an amorphous matrix (Figure 3i–l) and enriched in F, suggesting a salt anion (PF_6_^−^)‐derived SEI (Figures S5–S7 and Table S2). Elementally, O is uniformly distributed throughout the SEI, while C, P, and F are more concentrated in the outer layer (Figure S6d).

### 2.3 Disparity of SEI Layer on Cu Governs Li Morphology

We have clearly demonstrated that tuning electrolyte chemistry enables control of Li morphology. In LHCE, Li deposition is predominantly in the form of particles, with a minor fraction as whiskers. Conversely, in LCE, Li whiskers dominate, with particles as a minor fraction. In all cases, the SEI on Li particles is dominantly derived from salt anions, while the SEI on Li whiskers is mainly solvent‐derived. These results indicate that the differences in SEI composition to determine Li morphology.

**FIGURE 4:**
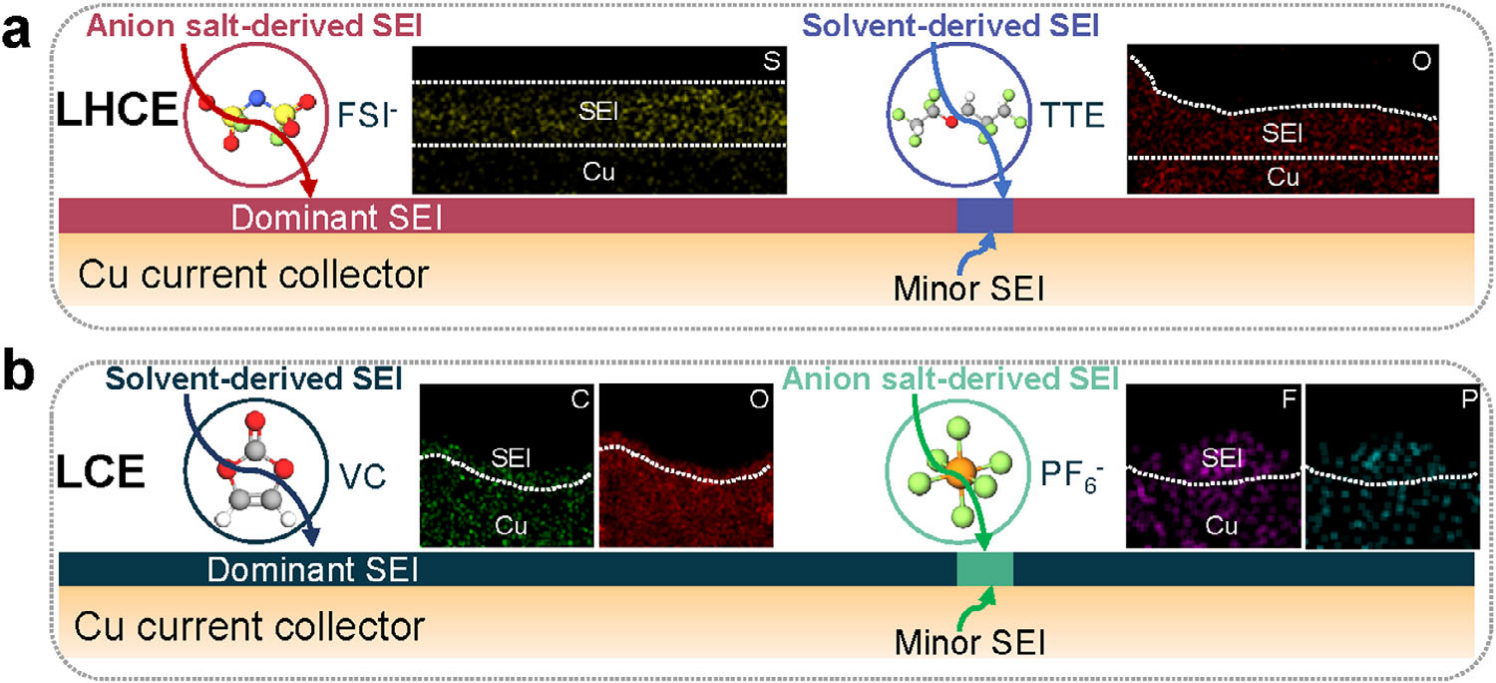
Characteristics of SEI layer on Cu current collector. (a) SEI formed on Cu current collector in LCE is dominated by solvent derived, but with small fraction of salt anion derived SEI (minor SEI). (b) SEI formed on Cu current collector in LHCE is dominated by salt anion derived, but with small fraction of solvent derived SEI (minor SEI).

Importantly, we reveal that this disparity in Li morphology originates from variations in the initial SEI layer formed on Cu (SEI_Cu_), which serves as the foundation for Li deposition. Under applied overpotential (from open circuit voltage (OCV) to 0 V), partial electrolyte decomposition on the Cu surface forms SEI_Cu_ [24, 25]. For LHCE, SEI_Cu_ is predominantly rich in S and low in O, indicating that its formation is dominated by FSI^−^ anion decomposition (Figure 4a; Figure S8a,b, Figure S9, and Table S3), similar to the SEI found on Li particles. A minor fraction of SEI_Cu_ is enriched with C and O (Figure 4a; Figure S8c,d, Figure S9, and Table S3), resembling the SEI on Li whiskers. In contrast, for LCE, SEI_Cu_ is mainly rich in C, suggesting a solvent‐derived origin (Figure 4b; Figures S10a,b, and S11, and Table S4), while a small fraction is enriched with F, indicating a contribution from anion salt decomposition (Figure 4b; Figure S10c,d, and S11, and Table S4).

These findings clarify that, depending on the electrolyte, SEI_Cu_ composition is either solvent‐ or salt‐anion dominant. Solvent‐derived organic SEI_Cu_ favors the formation of Li whiskers, whereas salt‐anion‐derived inorganic SEI_Cu_ favors Li particles. Deviations from the dominant SEI_Cu_ composition lead to minority Li morphologies.

A critical question remains: what governs the alienation of SEI_Cu_ within the same electrolyte? One plausible hypothesis is that the surface defects, grain boundaries, and twin boundaries on the Cu current collector induce non‐uniform electric fields. These field gradients may, in turn, lead to SEI_Cu_ composition variations, ultimately influencing Li deposition morphology [25]. Clearly, a more systematic and mechanistic investigation is needed to fully understand this phenomenon.

### 2.4 Mechanical Properties of SEI Layers

Direct measurement of SEI mechanical properties is challenging as recently reviewed by Li et al. [14]. However, several indirect indicators allow us to infer them. The mechanical properties of organic and inorganic materials are closely linked to their response under electron and photon irradiation [26, 27, 28]. In closely controlled cryo‐TEM experiments, we observed that SEI layers on Li whiskers and particles exhibit different degrees of electron beam sensitivity. In both cases, the outer SEI layer was more susceptible to beam damage, consistent with the widely accepted two‐layered structure: an inorganic‐dominated inner layer and an organic‐rich outer layer [29]. Since inorganic Li‐containing species generally has higher threshold displacement energies than organic components [30], the observed beam sensitivity provides insight into the relative mechanical properties of different SEI chemistries.

**FIGURE 5:**
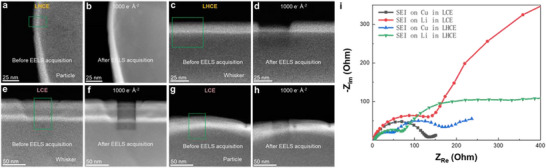
Physical properties of SEI. Cryo‐STEM image indicating SEI on Li whisker is more susceptible to electron beam damage than on Li particle. Electrochemical impedance spectra show lower impedance of SEI on Li particle. In LHCE system: (a) Cryo‐STEM image of SEI layer on Li particle, where the green marked region is mapped with EELS. (b) Cryo‐STEM image of SEI layer after the EELS mapping, showing no obvious beam damage. (c) Cryo‐STEM image of SEI layer on Li whisker, where the green marked region is mapped with EELS. (d) Cryo‐STEM image of SEI layer after the EELS mapping, indicating significant beam damage. In LCE system: (e) Cryo‐STEM image of SEI layer on Li whisker, where the green marked region is mapped with EELS. (f) Cryo‐STEM image of SEI layer after the EELS mapping, indicating significant beam damage. (g) Cryo‐STEM image of SEI layer on Li particle, where the green marked region is mapped with EELS. (h) Cryo‐STEM image of SEI layer after the EELS mapping, showing no obvious beam damage. (i) Nyquist plots comparing the impedance evolution of SEI on Cu and Li deposits formed in LHCE and LCE electrolytes.

The SEI layer on Li particle deposited either in LHCE (Figure 5a,b) or in LCE (Figure 5g,h) is not sensitive to electron beam. In contrast, the SEI on Li whiskers in the ether‐based LHCE (Figure 5c,d) and in the carbonate‐based LCE (Figure 5e,f), consistently displayed high sensitivity to electron beam. The SEI layer on Li whiskers was consistently more beam‐sensitive than on Li particles, suggesting lower modulus and fracture strength for the SEI on whiskers. Furthermore, the S‐rich outer layer of the anion‐derived SEI on Li particles in LHCE may enhance ductility, providing a potential explanation for its greater beam stability compared to the SEI on Li whiskers [31, 32, 33, 34]. These observations establish a direct correlation of mechanical properties of SEI layer with its composition and electron beam sensitivity.

It should be noted that atomic force microscopy (AFM)‐based nanoindentation/force spectroscopy techniques can be used to quantitatively measure the mechanical properties of SEI layer [14]. This represents a promising direction for future detailed investigation, particularly for the SEI formed on Li whiskers and Li particles within the same electrolyte environment.

### 2.5 Ionic Conductivity of the SEI Layers

We evaluated the ionic conductivity of SEI layers on both Cu and Li using two approaches: electrochemical impedance spectroscopy (EIS) measurement and composition/phase analysis.

EIS measurements of SEI layers on Cu and Li are shown in Figure 5i and Figure S12. The impedance follows the order: SEI_Cu_ (LHCE) < SEI_Li_ (LHCE) < SEI_Cu_ (LCE) < SEI_Li_ (LCE), indicating the corresponding ionic conductivity sequence: SEI_Cu_ (LHCE) > SEI_Li_ (LHCE) > SEI_Cu_ (LCE) > SEI_Li_ (LCE). These EIS results align well with the inferred ionic conductivity based on SEI composition and phase. In the ether‐based LHCE, SEI_Cu_ is rich in S, which is known to confer high ionic conductivity [31, 32, 33, 34]. In particular, the Li^+^ migration barrier in a Li_2_S‐modified interfacial layer is lower than that in a LiF‐modified interfacial layer [32], further supporting the higher conductivity observed.

It is worth noting that ionic conductivity (σ) is often estimated from ohmic resistance measurements using the relation: σ = t/(R × A), where t is the thickness of the SEI layer, R is the ohmic resistance, and A is the cross‐sectional area of the measurement. However, it is important to recognize that the measured resistance R is generally a convolution of electronic and ionic contributions. Because electronic carriers typically relax much faster than ionic species, the measured resistance is often dominated by the electronic component, especially under steady state or low‐frequency conditions. Indeed, this issue was recently highlighted by Xu et al. [18] who concluded that their measured ohmic resistance across the SEI layer primarily reflects electronic conductivity, rather than ionic conductivity.

### 2.6 Phase Field Modeling of Li Morphological Evolution

To elucidate how the chemomechanical properties of SEI influence Li morphology, we developed a phase‐field model to investigate the effects of ionic conductivity and Young's modulus of the SEI layers on Li deposition (Supplementary Information). The model incorporates an order parameter to represent the Li metal phase, together with three coupled fields: the Li^+^ concentration field to track Li deposition, the electric potential to capture electrochemical driving forces, and the displacement field to simulate stress evolution during dendrite growth. Within this framework, a free energy functional is constructed and the governing equations are formulated, enabling simultaneous simulations of Li^+^ transport, Li dendrite growth, electric field distribution, and stress evolution [35].

**FIGURE 6:**
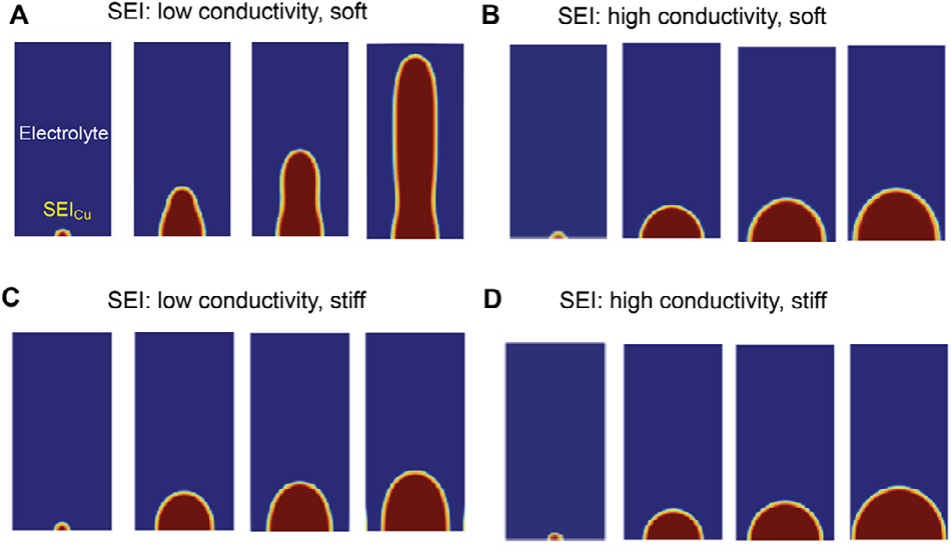
Phase‐field modeling of the effect of ionic conductivity and mechanical properties of SEI layers on the disparate Li growth morphologies. (a) SEI layer with low ionic conductivity and high stiffness, leading to particle growth. (b) SEI layer with low ionic conduction and low stiffness, leading to whisker growth. (c) SEI layer with high ionic conduction and high stiffness, leading to particle growth. (d) SEI layer with high ionic conduction and low stiffness, leading to whisker growth. Blue: electrolyte; yellow: SEI layer; red: Li metal.

During electrodeposition, Li^+^ ions migrate from the bulk liquid electrolyte through the SEI layer to the SEI/Li interface, where they combine with electrons to form new Li metal. Continuous Li deposition at the interface generates volumetric strain, which effectively stretches the surrounding SEI layer, leading to stress accumulation [36, 37]. Both Li^+^ conductivity and mechanical stiffness of the SEI layer strongly influence the electrodeposition process. Experimental study indicates that the SEI on Li whiskers is mechanically softer than that on Li particles, and that SEI_Cu_ (LCE) exhibits lower ionic conductivity than SEI_Cu_ (LHCE). Consistent with these observations, our simulations show that the combination of low modulus with low ionic conductivity favors whisker growth, whereas higher ionic conductivity or higher modulus leads particle‐like morphologies. Only when the SEI is both soft and poorly conductive do dendrites develop into whiskers (Figure 6).

The growth morphology is governed by the interplay of ionic conductivity and mechanical confinement [38, 39], as illustrated in Figures S13–S15. Due to lithium's high electronic conductivity, the tip of a growing Li dendrite experiences an intensified local electric field. The field concentration promotes Li^+^ accumulation toward the tip, establishing a tip‐base asymmetry that favors whisker‐like growth [40]. However, when the ionic conductivity of surrounding medium is high, Li^+^ ions can fast redistribute more evenly along the SEI/Li interface (Figure S13). This lateral ion transport leads to a more spatially uniform electrochemical driving force and supports the formation of isotropic, particle‐like Li deposits rather than whiskers.

Mechanical confinement further modulates the growth morphology by altering the local pressure environment during Li deposition [36, 37]. A mechanically stiffer SEI layer exerts stronger confinement to the growing Li deposits, generating higher hydrostatic pressure at the deposition front (Figure S14). This pressure modifies the local deposition kinetics through the pressure‐dependent Butler–Volmer relationship [35, 41], thereby inhibiting localized deposition. Since the hydrostatic pressure is highest at the dendrite tip, the effect of pressure is the highest therein, effectively suppressing tip propagation and promoting a more equiaxed, particle‐like growth morphology. In a more general term, local geometry at the interface is a key factor to influence local stress concentration, which correspondingly plays a regulatory role in Li deposition morphology [42, 43].

## 3 Conclusion

Across carbonate‐ and ether‐based electrolytes, Li morphology ranges from particles to whiskers. We reveal that morphology correlates with the structure and chemistry of the SEI layer, which are governed by electrolyte formulation. Specifically, an SEI that is ionically conductive and mechanically stiff promotes uniform interfacial kinetics and particle‐like deposition, whereas an SEI that is ionically resistive and mechanically soft concentrates ionic flux at protrusions and drives whisker growth. Hence, electrolyte design reduces to two measurable targets: ionic conductivity and stiffness of SEIs; either is a promising route to inhibit Li whiskers, suggesting transferability to other metal anodes and multiphase SEIs.

## 4 Experimental Section

### 4.1 Electrochemistry

In an Ar‐filled glove box, CR2032 coin‐cells were assembled with Cu TEM grid on a Cu foil as the working electrode and Li metal as the counter electrode and reference electrodes. A polyethylene separator was used to separate the two electrodes. Traditional carbonate‐based electrolyte 1.2 m LiPF_6_ in EC/EMC (3:7 by weight) with 5 wt.% VC and localized high concentration electrolyte (LHCE) composed of lithium bis(fluorosulfonyl)imide (LiFSI) and 1,2‐dimethoxyethane (DME) by adding 1,1,2,2‐tetrafluoroethyl‐2,2,3,3‐tetrafluoropropylether (TTE) as the diluent at a molar ratio of 1:1.2:3 were prepared in the glove box. Li metal was deposited onto the working electrode at a current density of 0.1 mA cm^−2^ for 100 min (by using Arbin BT‐2000).

### 4.2 Electrochemical Impedance Spectroscopy (EIS)

EIS was tested after open circuit voltage down to 0 V to acquire the impedance of SEI layer formed on Cu, and after deposition of Li metal onto the working electrode to acquire the impedance of SEI layer formed on Li. EIS measurements were conducted on a 1255B Solartron frequency response analyzer and a 1287 Solartron electrochemical workstation in the frequency range from 100 kHz to 10 mHz and amplitude of 5 mV.

### 4.3 Cryo‐transfer Procedure

After deposition, the TEM half grid was taken out from the coin cell and slightly rinsed with EMC to remove trace electrolyte and Li salts in the glovebox. Then, the TEM half grid with SEI formed on was placed in a sealed bag fulfilled with Ar. The sealed bag was plunged directly into a bath of liquid nitrogen after taken from the Ar‐filled glove box until the TEM half grid reached to very low temperature (around 100 k). We then quickly took out the Cu TEM half grid from the sealed bag and loaded it onto a pre‐cooling Gatan cryo‐holder (Elsa, Gatan, USA) using a cryo‐transfer station to ensure the entire process occurred under cryogenic environment.

### 4.4 Cryo‐TEM Characterization of the Electrochemical Deposited Li and SEI

Cryo‐TEM observations were performed on a 300 kV FEI Titan monochromated (scanning) transmission electron microscope ((S)TEM) equipped with a probe aberration corrector. The samples were viewed at low temperature (100 K) under low dose condition (∼100 e·Å^−2^·s^−1^ for high‐resolution TEM imaging). Energy dispersive X‐ray spectroscopy (EDS) elemental mapping was collected by scanning the same region multiple times at a dwell time of 0.01 ms and electron probe current ∼40 pA. Electron energy loss spectrums (EELS) were collected on a Gatan GIF‐Quantum spectrometer. The EELS collection semi‐angle during the spectroscopy experiments was ∼45 mrad. Each map was acquired in Dual EELS mode, with both low‐loss and high‐loss regions of the spectrum recorded. The low‐loss maps included the zero‐loss peak, the position of which was used to align the energy axis of the low‐ and high‐loss regions of the spectra simultaneously, resulting in a flat energy surface across the map. EELS spectra dispersion was 0.25 eV per channel with vertical binning as 130. The probe beam current was around 25 pA, pixel dwell time as 0.01–0.05s. The electron dose applied during the acquisition of the EELS spectra shown in the main text was 5–500 e^−^ Å^−2^. All those electron dose rates did not introduce obvious damages after acquired images, EDS and EELS spectrum [20, 21, 44].

It should be pointed out that, in analyzing the composition of SEI layer on Li with different morphologies, such as Li whiskers and particles within the same cell, high spatial resolution is essential to accurately capture compositional differences. Unfortunately, large‐area techniques like X‐ray photoelectron spectroscopy (XPS) lack the spatial resolution necessary to distinguish between the SEI on a Li whisker versus that on a Li particle within the same cell. In contrast, EDS in STEM provides nanoscale resolution, enabling a direct correlation between SEI composition and a specific local morphology within the same cell. With the high collection angle EDS system in STEM, the typical error is around 0.2%, allowing for confident and localized chemical analysis of the SEI layer in heterogeneous Li morphologies.

## Author Contributions

C.W., Y.X., S. Z. designed the work. Y.X. collected, analyzed experimental data for cryo‐TEM studies, and drafted the manuscript under the direction of C.W. and W.X., D.X., R.F. and S.Z. performed phase field simulations. H.J. and P.L. performed the electrochemical measurements. All authors edited and finalized the manuscript.

## Conflicts of Interest

The authors declare no conflicts of interest.

## Supporting information




**Supporting File**: adma72564‐sup‐0001‐SuppMat.docx.

## Data Availability

The data that support the findings of this study are available from the corresponding author upon reasonable request.

## References

[1] M. S. Dresselhaus and I. L. Thomas , “Alternative Energy Technologies,” Nature 414 (2001): 332–337, 10.1038/35104599. 11713539

[2] B. Dunn , H. Kamath , and J. M. Tarascon , “Electrical Energy Storage for the Grid: A Battery of Choices,” Science 334 (2011): 928–935, 10.1126/science.1212741. 22096188

[3] M. Li , J. Lu , Z. W. Chen , and K. Amine , “30 Years of Lithium‐Ion Batteries,” Advanced Materials 30, no. 33 (2018): 1800561, 10.1002/adma.201800561. 29904941

[4] J. Liu , Z. N. Bao , Y. Cui , et al., “Pathways for Practical High‐Energy Long‐Cycling Lithium Metal Batteries,” Nature Energy 4, no. 3 (2019): 180–186, 10.1038/s41560-019-0338-x.

[5] J. B. Goodenough and Y. Kim , “Challenges for Rechargeable Li Batteries,” Chemistry of Materials 22, no. 3 (2010): 587–603, 10.1021/cm901452z.

[6] K. Xu , “Electrolytes and Interphases in Li‐Ion Batteries and Beyond,” Chemical Reviews 114, no. 23 (2014): 11503–11618, 10.1021/cr500003w. 25351820

[7] X. B. Cheng , R. Zhang , C. Z. Zhao , and Q. Zhang , “Toward Safe Lithium Metal Anode in Rechargeable Batteries: A Review,” Chemical Reviews 117, no. 15 (2017): 10403–10473, 10.1021/acs.chemrev.7b00115. 28753298

[8] D. C. Lin , Y. Y. Liu , and Y. Cui , “Reviving the Lithium Metal Anode for High‐Energy Batteries,” Nature Nanotechnology 12, no. 3 (2017): 194–206, 10.1038/nnano.2017.16. 28265117

[9] C. Fang , J. Li , M. Zhang , et al., “Quantifying Inactive Lithium in Lithium Metal Batteries,” Nature 572, no. 7770 (2019): 511–515, 10.1038/s41586-019-1481-z. 31435056

[10] J. Zheng , M. S. Kim , Z. Tu , S. Choudhury , T. Tang , and L. A. Archer , “Regulating Electrodeposition Morphology of Lithium: Towards Commercially Relevant Secondary Li Metal Batteries,” Chemical Society Reviews 49, no. 9 (2020): 2701–2750, 10.1039/C9CS00883G. 32232259

[11] P. Zou , Y. Sui , H. Zhan , et al., “Polymorph Evolution Mechanisms and Regulation Strategies of Lithium Metal Anode Under Multiphysical Fields,” Chemical Reviews 121, no. 10 (2021): 5986–6056, 10.1021/acs.chemrev.0c01100. 33861070

[12] M. J. Hossain , Q. Wu , E. J. Marin Bernardez , et al., “The Relationship Between Ionic Conductivity and Solvation Structures of Localized High‐Concentration Fluorinated Electrolytes for Lithium‐Ion Batteries,” The Journal of Physical Chemistry Letters 14 (2023): 7718–7731, 10.1021/acs.jpclett.3c01453. 37606601

[13] M. Werres , D. Niedziela , A. Latz , and B. Horstmann , “Stress‐Driven Whisker Formation in Lithium Metal Batteries,” Nano Letters 25, no. 29 (2025): 11244–11250, 10.1021/acs.nanolett.5c01910. 40657676 PMC12291588

[14] J. Li , Y. Wang , S. Sun , et al., “Understanding and Regulating the Mechanical Stability of Solid Electrolyte Interphase in Batteries,” Advanced Energy Materials 15, no. 4 (2025): 2403845.

[15] Y. Liu , X. Xu , O. O. Kapitanova , et al., “Electro‐Chemo‐Mechanical Modeling of Artificial Solid Electrolyte Interphase to Enable Uniform Electrodeposition of Lithium Metal Anodes,” Advanced Energy Materials 12, no. 9 (2022): 2103589.

[16] A. Pei , G. Y. Zheng , F. F. Shi , Y. Z. Li , and Y. Cui , “Nanoscale Nucleation and Growth of Electrodeposited Lithium Metal,” Nano Letters 17, no. 2 (2017): 1132–1139, 10.1021/acs.nanolett.6b04755. 28072543

[17] E. Peled and S. Menkin , “Review—SEI: Past, Present and Future,” Journal of The Electrochemical Society 164, no. 7 (2017): A1703–A1719, 10.1149/2.1441707jes.

[18] Y. Xu , H. Jia , P. Gao , et al., “Direct In Situ Measurements of Electrical Properties of Solid–Electrolyte Interphase on Lithium Metal Anodes,” Nature Energy 8, no. 12 (2023): 1345–1354, 10.1038/s41560-023-01361-1. 38249622 PMC10798234

[19] X. D. Ren , L. F. Zou , X. Cao , et al., “Enabling High‐Voltage Lithium‐Metal Batteries under Practical Conditions,” Joule 3 (2019): 1662–1676.

[20] Y. Xu , H. Wu , Y. He , et al., “Atomic to Nanoscale Origin of Vinylene Carbonate Enhanced Cycling Stability of Lithium Metal Anode Revealed by Cryo‐Transmission Electron Microscopy,” Nano Letters 20, no. 1 (2020): 418–425, 10.1021/acs.nanolett.9b04111. 31816244

[21] Y. Xu , H. Wu , H. Jia , J. G. Zhang , W. Xu , and C. Wang , “Current Density Regulated Atomic to Nanoscale Process on Li Deposition and Solid Electrolyte Interphase Revealed by Cryogenic Transmission Electron Microscopy,” ACS Nano 14, no. 7 (2020): 8766–8775, 10.1021/acsnano.0c03344. 32598126

[22] D. Aurbach , Y. Einely , and A. Zaban , “The Surface Chemistry of Lithium Electrodes in Alkyl Carbonate Solutions,” Journal of The Electrochemical Society 141, no. 1 (1994): L1–L3, 10.1149/1.2054718.

[23] E. Peled , D. Golodnitsky , and G. Ardel , “Advanced Model for Solid Electrolyte Interphase Electrodes in Liquid and Polymer Electrolytes,” Journal of The Electrochemical Society 144, no. 8 (1997): L208–L210, 10.1149/1.1837858.

[24] W. Huang , D. T. Boyle , Y. Z. Li , et al., “Nanostructural and Electrochemical Evolution of the Solid‐Electrolyte Interphase on CuO Nanowires Revealed by Cryogenic‐Electron Microscopy and Impedance Spectroscopy,” ACS Nano 13, no. 1 (2019): 737–744, 10.1021/acsnano.8b08012. 30589528

[25] Y. B. Xu , H. P. Wu , H. Jia , et al., “Sweeping Potential Regulated Structural and Chemical Evolution of Solid‐Electrolyte Interphase on Cu and Li as Revealed by Cryo‐TEM,” Nano Energy 76 (2020): 105040, 10.1016/j.nanoen.2020.105040.

[26] K. Zheng , C. C. Wang , Y. Q. Cheng , et al., “Electron‐Beam‐Assisted Superplastic Shaping of Nanoscale Amorphous Silica,” Nature Communications 1 (2010): 24, 10.1038/ncomms1021. PMC3047011 20975693

[27] Y. Oshima , A. Nakamura , and K. Matsunaga , “Extraordinary Plasticity of an Inorganic Semiconductor in Darkness,” Science 360, no. 6390 (2018): 772–774, 10.1126/science.aar6035. 29773747

[28] J. Ahmed , J. Wu , S. Mushtaq , and Y. Zhang , “Effects of Electron Beam Irradiation and Multi‐Functional Monomer/Co‐Agents on the Mechanical and Thermal Properties of Ethylene‐Vinyl Acetate Copolymer/Polyamide Blends,” Materials Today Communication 23 (2020): 100840.

[29] Y. Zhou , M. Su , X. Yu , et al., “Real‐Time Mass Spectrometric Characterization of the Solid–Electrolyte Interphase of a Lithium‐Ion Battery,” Nature Nanotechnology 15, no. 3 (2020): 224–230, 10.1038/s41565-019-0618-4. 31988500

[30] A. Jaberi , N. Brodusch , J. Song , and R. Gauvin , “Prediction of Primary Knock‐On Damage During Electron Microscopy Characterization of Lithium‐Containing Materials,” Ultramicroscopy 256 (2024): 113884, 10.1016/j.ultramic.2023.113884. 37976971

[31] H. Chen , A. Pei , D. C. Lin , et al., “Uniform High Ionic Conducting Lithium Sulfide Protection Layer for Stable Lithium Metal Anode,” Advanced Energy Materials 9, no. 22 (2019): 1900858, 10.1002/aenm.201900858.

[32] C. Lai , C. Y. Shu , W. Li , et al., “Stabilizing a Lithium Metal Battery by an In Situ Li_2_S‐modified Interfacial Layer via Amorphous‐Sulfide Composite Solid Electrolyte,” Nano Letters 20, no. 11 (2020): 8273–8281, 10.1021/acs.nanolett.0c03395. 33108209

[33] Y. G. Zhang , N. Du , and D. R. Yang , “Designing Superior Solid Electrolyte Interfaces on Silicon Anodes for High‐Performance Lithium‐Ion Batteries,” Nanoscale 11, no. 41 (2019): 19086–19104, 10.1039/C9NR05748J. 31538999

[34] J. G. Xu , H. K. Tian , J. Qi , Y. Qi , Q. L. Zhang , and X. C. Xiao , “Mechanical and Electronic Stabilization of Solid Electrolyte Interphase with Sulfite Additive for Lithium Metal Batteries,” Journal of The Electrochemical Society 166, no. 14 (2019): A3201–A3206, 10.1149/2.0331914jes.

[35] D. Xue , C. Fincher , R. Fang , B. W. Sheldon , L. Chen , and S. Zhang , “Dynamic Interplay of Dendrite Growth and Cracking in Lithium Metal Solid‐State Batteries,” Journal of the Mechanics and Physics of Solids 202 (2025): 106197, 10.1016/j.jmps.2025.106197.

[36] L. M. Suo , W. J. Xue , M. Gobet , et al., “Fluorine‐Donating Electrolytes Enable Highly Reversible 5‐V‐class Li Metal Batteries,” Proceedings of the National Academy of Sciences 115, no. 6 (2018): 1156–1161, 10.1073/pnas.1712895115. PMC5819397 29351993

[37] Y. He , X. D. Ren , Y. B. Xu , et al., “Origin of Lithium Whisker Formation and Growth Under Stress,” Nature Nanotechnology 14, no. 11 (2019): 1042–1047, 10.1038/s41565-019-0558-z. 31611656

[38] X. Z. Guan , A. X. Wang , S. Liu , et al., “Controlling Nucleation in Lithium Metal Anodes,” Small 14 (2018): 1801423. 10.1002/smll.201801423 30047235

[39] X. R. Chen , B. C. Zhao , C. Yan , and Q. Zhang , “Review on Li Deposition in Working Batteries: From Nucleation to Early Growth,” Advanced Materials 33, no. 8 (2021): 2004128, 10.1002/adma.202004128. 33432664

[40] X. R. Chen , Y. X. Yao , C. Yan , R. Zhang , X. B. Cheng , and Q. Zhang , “A Diffusion–Reaction Competition Mechanism to Tailor Lithium Deposition for Lithium‐Metal Batteries,” Angewandte Chemie International Edition 59, no. 20 (2020): 7743–7747, 10.1002/anie.202000375. 32160379

[41] L. Zhang , T. Yang , C. Du , et al., “Lithium Whisker Growth and Stress Generation in an In Situ Atomic Force Microscope–Environmental Transmission Electron Microscope Set‐Up,” Nature Nanotechnology 15, no. 2 (2020): 94–98, 10.1038/s41565-019-0604-x. 31907440

[42] Y. Liu , X. Xu , X. Jiao , O. O. Kapitanova , Z. Song , and S. Xiong , “Role of Interfacial Defects on Electro–Chemo–Mechanical Failure of Solid‐State Electrolyte,” Advanced Materials 35, no. 24 (2023): 2301152, 10.1002/adma.202301152. 37060331

[43] J. Wu , R. Yao , K. Wang , et al., “Prior‐Knowledge‐Driven Machine Learning Modeling for Electro‐Chemo‐Mechanical Failure of Solid‐State Electrolyte,” Journal of Energy Chemistry 111 (2025): 119–128, 10.1016/j.jechem.2025.07.039.

[44] M. J. Zachman , Z. Tu , S. Choudhury , L. A. Archer , and L. F. Kourkoutis , “Cryo‐STEM Mapping of Solid–Liquid Interfaces and Dendrites in Lithium‐Metal Batteries,” Nature 560, no. 7718 (2018): 345–349, 10.1038/s41586-018-0397-3. 30111789

