# Supplementary material for: Chemomechanical Origin of Morphological Disparity in Lithium Metal Electrodeposition
Source: Adv Mater. 2026 Feb 15;38(16):e22026. doi: 10.1002/adma.202522026 (PMC12994307; doi:10.1002/adma.202522026)
Supplement: Supplementary file 1 — Supporting File: adma72564‐sup‐0001‐SuppMat.docx. [file ADMA-38-e22026-s001.docx]

Supplementary Materials for

**Chemomechanical origin of morphological disparity in lithium metal electrodeposition**

Yaobin Xu^1†*^, Ruyue Fang^2†^, Dingchuan Xue^2^, Hao Jia^1^, Phung M. L. Le^1^, Ji-Guang Zhang^1^, Wu Xu^1^, Sulin Zhang^2*^, Chongmin Wang^3*^

^1^Energy and Environment Directorate, Pacific Northwest National Laboratory, Richland, WA 99354, USA

^2^Department of Engineering Science and Mechanics, Pennsylvania State University, University Park, PA 16802, USA

^3^Environmental Molecular Sciences Laboratory, Pacific Northwest National Laboratory, Richland, WA 99354, USA

^†^These authors contributed equally to this work

* E-mail: [yaobin.xu@pnnl.gov](mailto:yaobin.xu@pnnl.gov), [suz10@psu.edu](mailto:suz10@psu.edu), [chongmin.wang@pnnl.gov](mailto:chongmin.wang@pnnl.gov)

**Phase field modeling of Li morphology**

To elucidate how the chemomechanical properties of SEI influence Li morphology, we develop a phase-field model to investigate the effects of ionic conductivity and Young’s modulus of the SEI on Li deposition. Following Xue et al.’s work,^1^ the model incorporates an order parameter $\xi\left( \boldsymbol{x},t \right)$ to represent the Li dendrite phase, together with three coupled fields: the Li^+^ concentration field $c\left( \boldsymbol{x},t \right)$ to track Li deposition, the electric potential $\phi\left( \boldsymbol{x},t \right)$ to capture electrochemical driving forces, and the displacement field $\boldsymbol{u}\left( \boldsymbol{x},t \right)$ to simulate stress evolution during dendrite growth. Within this framework, we construct the Helmholtz free energy functional.

$$\begin{aligned} \mathcal{F}\left( \xi,c,\boldsymbol{u},\phi\right)=\int\left( f_{\mathrm{chem}}+f_{\mathrm{int}}+f_{\mathrm{elec}}+f_{\mathrm{mech}} \right)dV\#\left( 1 \right) \end{aligned}$$

where $f_{\mathrm{chem}}$, $f_{\mathrm{int}}$, $f_{\mathrm{elec}}$, $f_{\mathrm{mech}}$ represent chemical, gradient, electrical, and mechanical energy densities. Here, the chemical energy density $f_{\mathrm{chem}}$ is expressed as

$$\begin{aligned} f_{\mathrm{chem}}\left( c \right)=\mu_{0}c+cRT\ln c\#\left( 2 \right) \end{aligned}$$

where $\mu_{0}$ is the standard chemical potential of Li^+^ in the electrolyte, $R$ is the gas constant and $T$ is the experiment temperature. The interfacial energy density between Li/Electrolyte is described as

$$\begin{aligned} f_{\mathrm{int}}\left( \xi\right)=W\xi^{2}\left( 1-\xi\right)^{2}+\frac{\kappa_{\xi}}{2}\left( \nabla\xi\right)^{2}\#\left( 3 \right) \end{aligned}$$

with barrier height $W$ and gradient coefficient $\kappa_{\xi}$. The two terms represent repulsion and adhesion, respectively, between Li and the electrolyte. The electric energy density is formulated as

$$\begin{aligned} f_{\mathrm{elec}}\left( \xi,c,\phi\right)=\left( cz_{\mathrm{Li}^{+}}+c_{B}z_{B} \right)F\phi\#\left( 4 \right) \end{aligned}$$

where $F$ is Faraday constant, $c_{B}$ is the density of total anions which is considered as immobile, and $z_{\mathrm{Li}^{+}}$ and $z_{B}$ represents the valance number of Li^+^ and anions.

The strain energy is described as

$$\begin{aligned} f_{\mathrm{mech}}\left( \xi,\boldsymbol{u} \right)=\frac{1}{2}\left( \boldsymbol{\varepsilon-}\boldsymbol{\varepsilon}^{0} \right)\mathbb{:C:}\left( \boldsymbol{\varepsilon-}\boldsymbol{\varepsilon}^{0} \right)\#\left( 5 \right) \end{aligned}$$

The elastic modulus tensor is expressed as $\mathbb{C}\left( \xi\right)=\psi_{\mathrm{Li}}\mathbb{C}_{\mathrm{Li}}+\psi_{\mathrm{SEI}}\mathbb{C}_{\mathrm{SEI}}+k$ where $\psi_{\mathrm{Li}}=h\left( \xi\right)-\psi_{\mathrm{SEI}}$ and $\psi_{\mathrm{SEI}}=h^{'}\left( \xi\right)/\left| h^{'}\left( \xi\right) \right|_{\max}$ are volume fractions of Li and SEI where $h\left( \xi\right)=6\xi^{5}-15\xi^{4}+10\xi^{3}$ is an interpolation function, and $k$ is a small number to avoid numerical issues in the liquid electrolyte phase. Here $\boldsymbol{\varepsilon}=(\nabla\boldsymbol{u+}\nabla\boldsymbol{u}^{\boldsymbol{\top}}\boldsymbol{)/}2$ is the total strain, and $\boldsymbol{\varepsilon}^{0}\boldsymbol{=}h\left( \xi\right)\varepsilon^{00}\boldsymbol{I}$ is the eigenstrain due to lithium dendrite growth.

Based on the above energy functional, we derived the corresponding governing equations for each field. The evolution of lithium metal phase is considered linearly proportional to the interfacial free energy and exponentially to the thermodynamics driving force related electrode reaction.^2, 3^

$$\begin{aligned} \frac{\partial\xi}{\partial t}=-L_{\xi}\mu_{\xi}+h^{'}\left( \xi\right)L_{\eta}R_{BV}, \#\left( 6 \right) \end{aligned}$$

where $L_{\xi}$ denotes the interfacial mobility and $L_{\eta}$ stands for the reaction constant. The first term in Eq. (2) describes the morphological changes of the deposited Li metal, arising from the driving force $\mu_{\xi}=\frac{\delta\mathcal{F}}{\delta\xi}=4W\xi\left( \xi-0.5 \right)\left( \xi-1 \right)-\kappa\nabla^{2}\xi$, while the second term accounts for the electrochemical reaction that occurs at the Li/Electrolyte interface, and the deposition rate is modelled by the Butler-Volmer kinetics: $R_{\mathrm{BV}}=\frac{c}{c_{0}}\exp\left( \frac{-\alpha F(\eta_{a}+\eta_{m})}{RT} \right)-\exp\left( \frac{\left( 1-\alpha\right)F\eta_{a}+\eta_{m}}{RT} \right).$^2, 4^ Here, $L_{\xi}$ and $L_{\eta}$ are the interface mobility and the reaction constant, respectively, and $\alpha$ is the symmetry factor. The saturated concentration of lithium in the electrolyte is $c_{0}$.The activation overpotential $\eta_{a}=\phi_{\mathrm{Li}}-\phi_{\mathrm{Electrolyte}}-E^{\theta}$ is the electrical potential drop at the Li/Electrolyte interface with $E^{\theta}$ as the standard half-cell potential, and the mechanical overpotential $\eta_{m}=\frac{pv_{\mathrm{Li}}}{F}$ is proportional to the hydrostatic pressure $p$ and molar volume of lithium metal $v_{\mathrm{Li}}$.

The migration of Li^+^ is driven by the electrochemical potential gradient $\nabla\mu_{c}$ where $\mu_{c}=\frac{\delta\mathcal{F}}{\delta c}=\mu_{0}+RT\left( 1+\ln c \right)+F\phi$, therefore governed by chemical potential gradient, electric potential gradient, and consumption due to Li deposition.

$$\begin{aligned} \frac{\partial c}{\partial t}=\nabla\cdot\left[ D_{c}\nabla c+D_{c}\frac{Fc}{RT}\nabla\phi\right]-c_{s}\frac{\partial\xi}{\partial t}\#\left( 7 \right) \end{aligned}$$

where $c_{s}$ is the site density of Li metal. Here, the Li^+^ diffusivity $D_{c}$ is described as $\psi_{\mathrm{Li}}D_{c}^{\mathrm{LM}}+\psi_{\mathrm{SEI}}D_{c}^{\mathrm{SEI}}+\left( 1-\psi_{\mathrm{Li}}-\psi_{\mathrm{SEI}} \right)D_{c}^{E}$ where $D_{c}^{\mathrm{LM}}$, $D_{c}^{\mathrm{SEI}}$ and $D_{c}^{E}$ represent the ionic diffusivities of Li metal, SEI, and the electrolyte. This equation is essentially the conservation of current density.^2^

Within the current framework, we impose the Poisson equation and mechanics equilibrium ($\delta\mathcal{F/}\delta\boldsymbol{u}=0$) over the simulation domain:

$$\begin{aligned} \nabla\cdot\left( \varepsilon_{\phi}\nabla\phi\right)=-F\left( cz_{\mathrm{Li}^{+}}+c_{B}z_{B} \right)\#\left( 8 \right) \end{aligned}$$

$$\begin{aligned} \nabla\cdot\boldsymbol{\sigma}=\boldsymbol{0}\#\left( 9 \right) \end{aligned}$$

Here, the stress tensor $\boldsymbol{\sigma}$ follows the linear elasticity constitutive law $\boldsymbol{\sigma}\mathbb{=C:(}\boldsymbol{\varepsilon-}\boldsymbol{\varepsilon}^{0}\boldsymbol{)}$ and the permittivity $\varepsilon_{\phi}$ is expressed as $h\left( \xi\right)\varepsilon_{\phi}^{\mathrm{LM}}+[1-h\left( \xi\right)]\varepsilon_{\phi}^{E}$ where $\varepsilon_{\phi}^{\mathrm{LM}}$ and $\varepsilon_{\phi}^{E}$ are permittivities of Li metal and electrolyte.

Our numerical simulation was implemented in COMSOL Multiphysics 6.2 and the corresponding computational parameters are listed in **Table S5**. We choose $D_{c}^{\mathrm{SEI}}=0.1D_{c}^{\mathrm{LM}}/10D_{c}^{\mathrm{LM}}$ to represent the low/high ionic conductivities, and $E_{\mathrm{SEI}}=0.1E_{\mathrm{Li}}/10E_{\mathrm{Li}}$ to represent the soft/stiff SEIs.

**
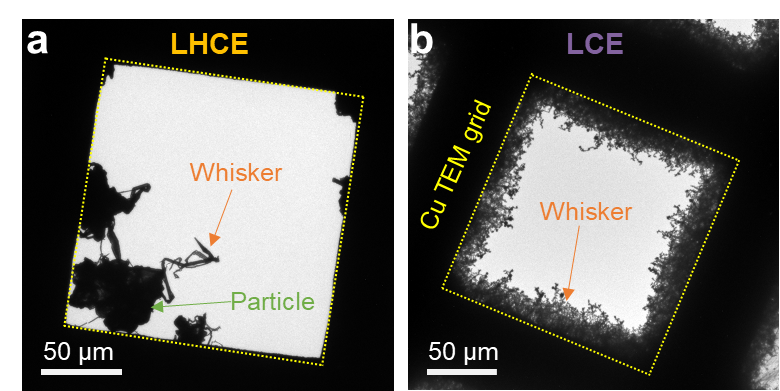
**

**Figure S1. Low magnification TEM images of Li deposits formed in different electrolyte. a.** LHCE and **b.** LCE.


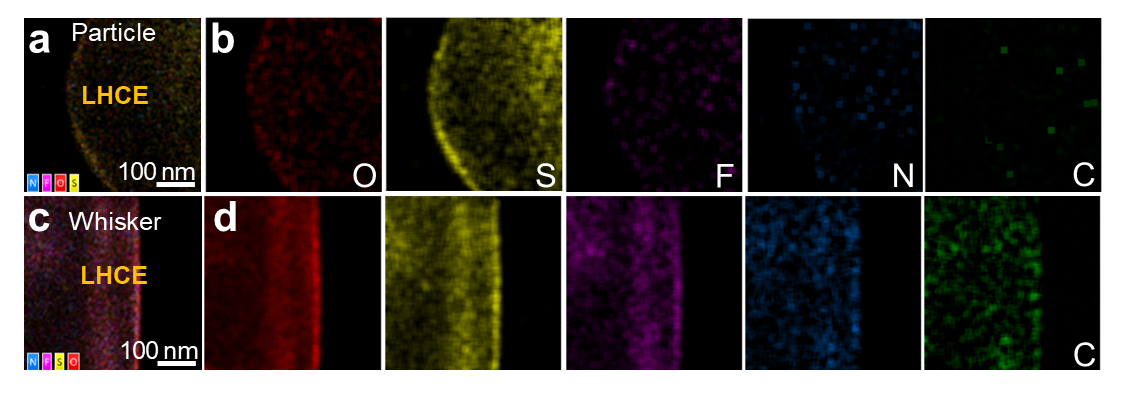


**Figure S2. Composition of SEI layers formed in the ether-based LHCE electrolyte at the current density of 0.1 mA cm^-2^.** The overlapped EDS image and the corresponding EDS maps of O, S, F, N, and C showing the elemental distribution of SEI on the **a**-**b.** Li particle and **c**-**d.** Li whisker formed in the ether-based LHCE.

**Figure S3. Composition of SEI layers in Li particle and Li whisker formed in the ether-based LHCE.**

**
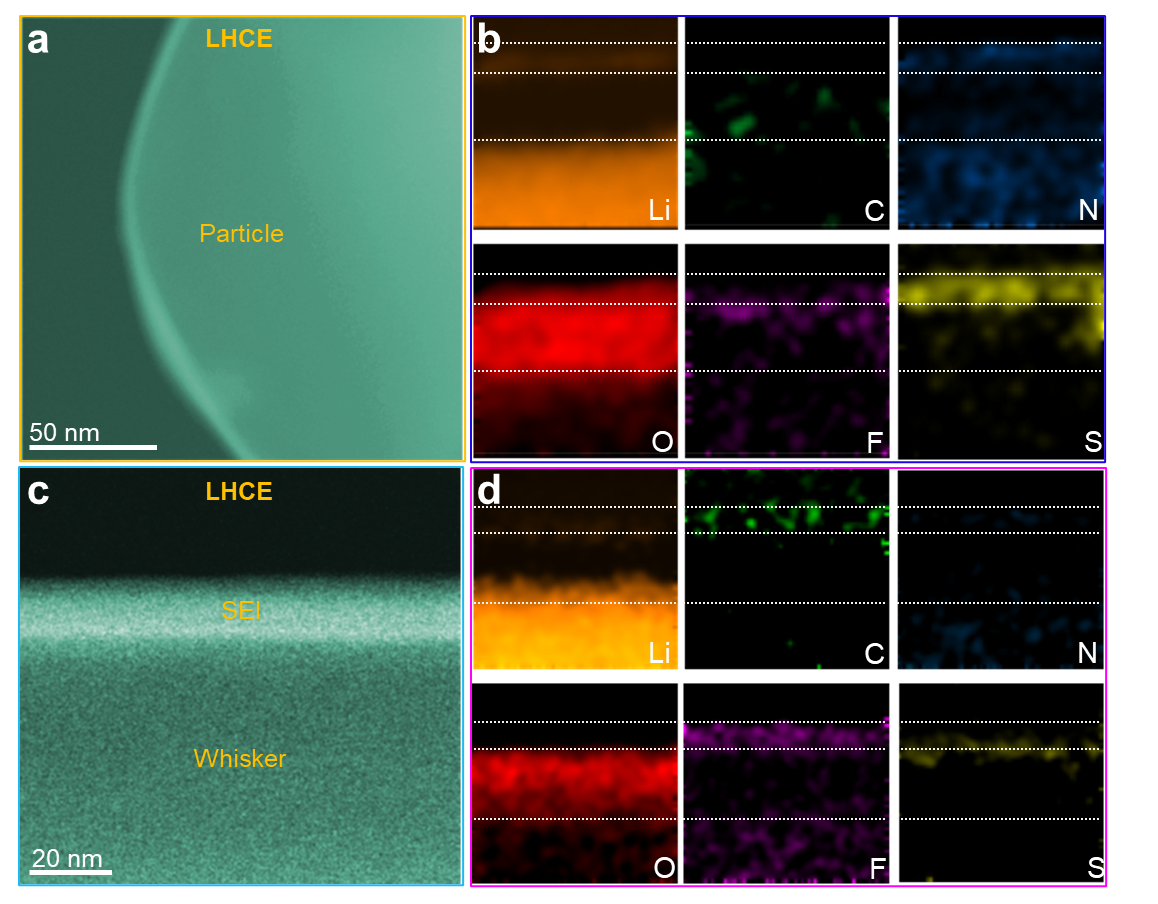
**

**Figure S4. Structure and composition of Li deposits and their SEIs. a.** Cryo-HAADF-STEM imaging reveals the SEI layer on the Li particle deposit formed in the ether-based LHCE. **b.** EELS elemental mapping showing the bilayer structure of SEI, the inner layer is oxygen-rich, the outer layer is sulfur-rich. **c.** Cryo-HAADF-STEM imaging reveals the SEI layer on the Li whisker deposit formed in the ether-based LHCE. **d.** EELS elemental mapping showing the bilayer structure of SEI, the inner layer is oxygen-rich, the outer layer is carbon and fluorine-rich.

**
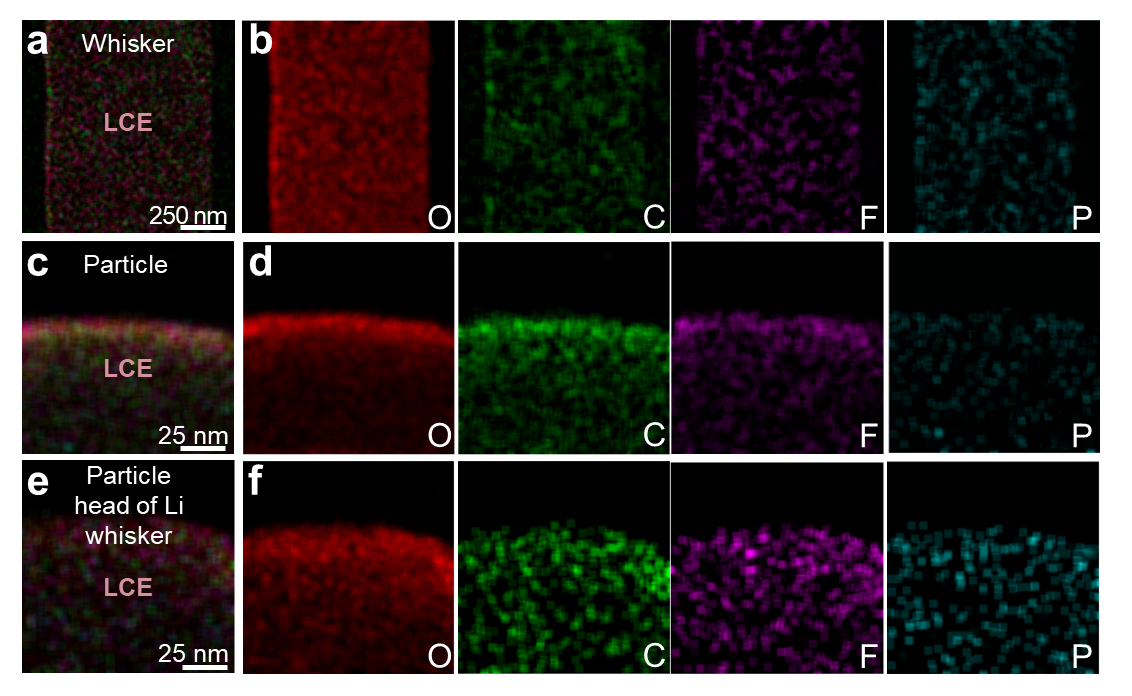
**

**Figure S5. Composition of SEI layers formed in the carbonate-based LCE at the current density of 0.1 mA cm^-2^.** The overlapped EDS image and the corresponding EDS maps of O, C, F, and P showing the elemental distribution of SEI on: **a-b.** Li whisker**, c-d.** Li particle, and **e-f.** Li particle head of a Li whisker formed in the carbonate-based LCE.

**
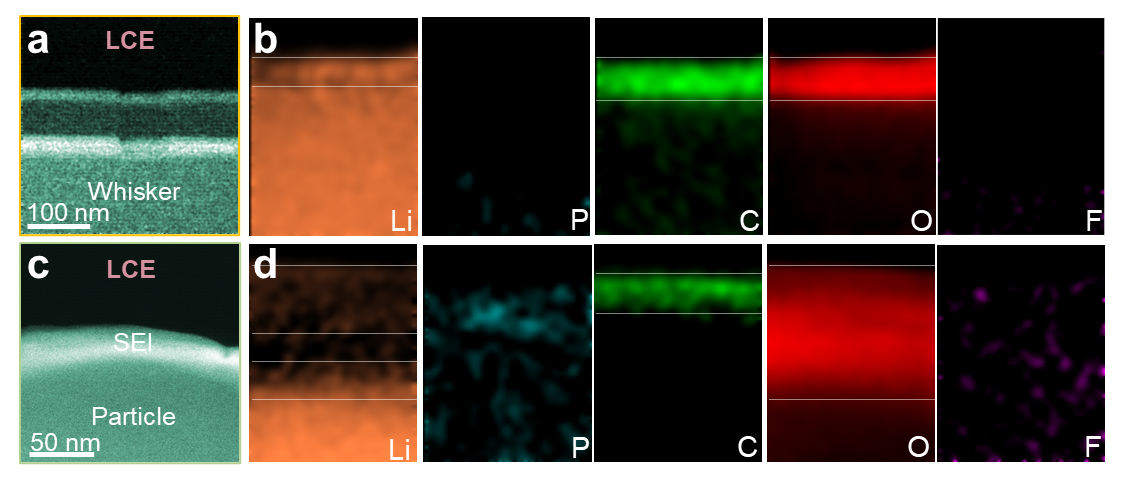
**

**Figure S6. Structure and elemental composition of Li deposits and their SEIs. a.** Cryo-HAADF-STEM imaging reveals the SEI layer on the Li whisker deposit formed in the carbonate-based LCE. **b.** EELS elemental mapping showing the monolithic structure of SEI. **c.** Cryo-HAADF-STEM imaging reveals the SEI layer on the Li particle deposit formed in the carbonate-based LCE. **d.** EELS elemental mapping showing the bilayer structure of SEI, the inner layer is oxygen-rich, the outer layer is carbon-rich.

**Figure S7. Composition of SEI layers in Li particle and Li whisker formed in carbonate-based LCE.**

**
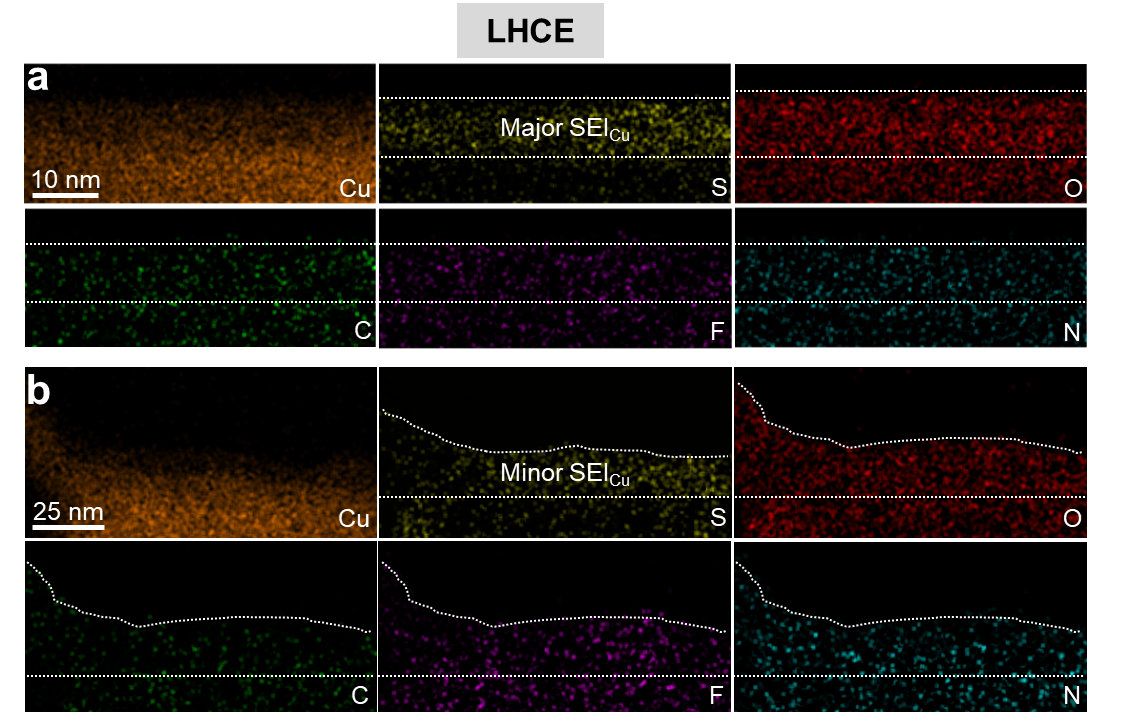
**

**Figure S8. Composition of SEI layers on the Cu foil formed in the ether-based LHCE.** The overlapped EDS image and the corresponding EDS maps of Cu, S, O, C, F, and P showing the elemental distribution of **a.** major and **b.** minor SEI on the Cu foil, which indicates the nonuniform of SEI distribution on the Cu foil formed in ether-based LHCE.

**Figure S9. Composition of SEI layers on the Cu current collector formed in the ether-based LHCE.**


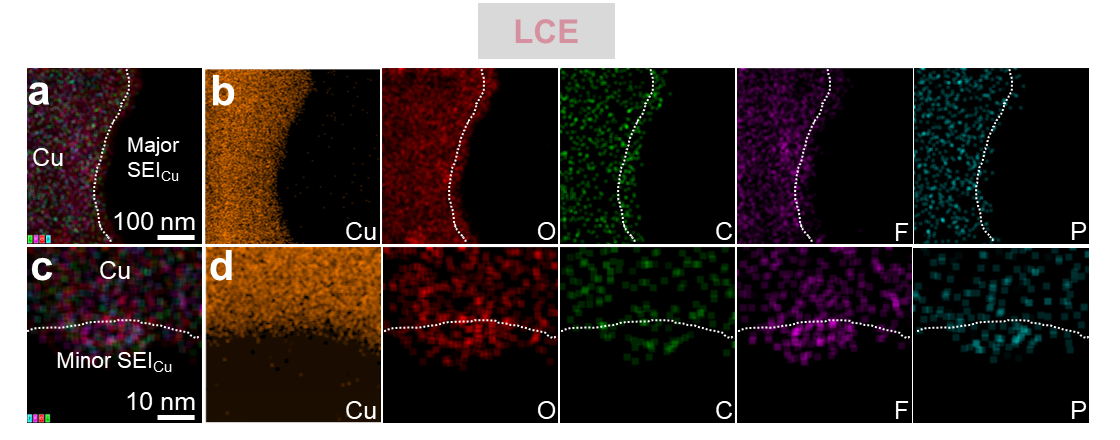


**Figure S10. Composition of SEI layers on the Cu foil formed in the carbonate-based LCE.** The overlapped EDS image and the corresponding EDS maps of Cu, O, C, F, and P showing the elemental distribution of **a-b.** major and **c-d.** minor SEI on the Cu foil, which indicates the nonuniform of SEI distribution on the Cu foil formed in carbonate-based LCE.

**Figure S11. Composition of SEI layers on the Cu current collector formed in the carbonate-based LCE.**

**
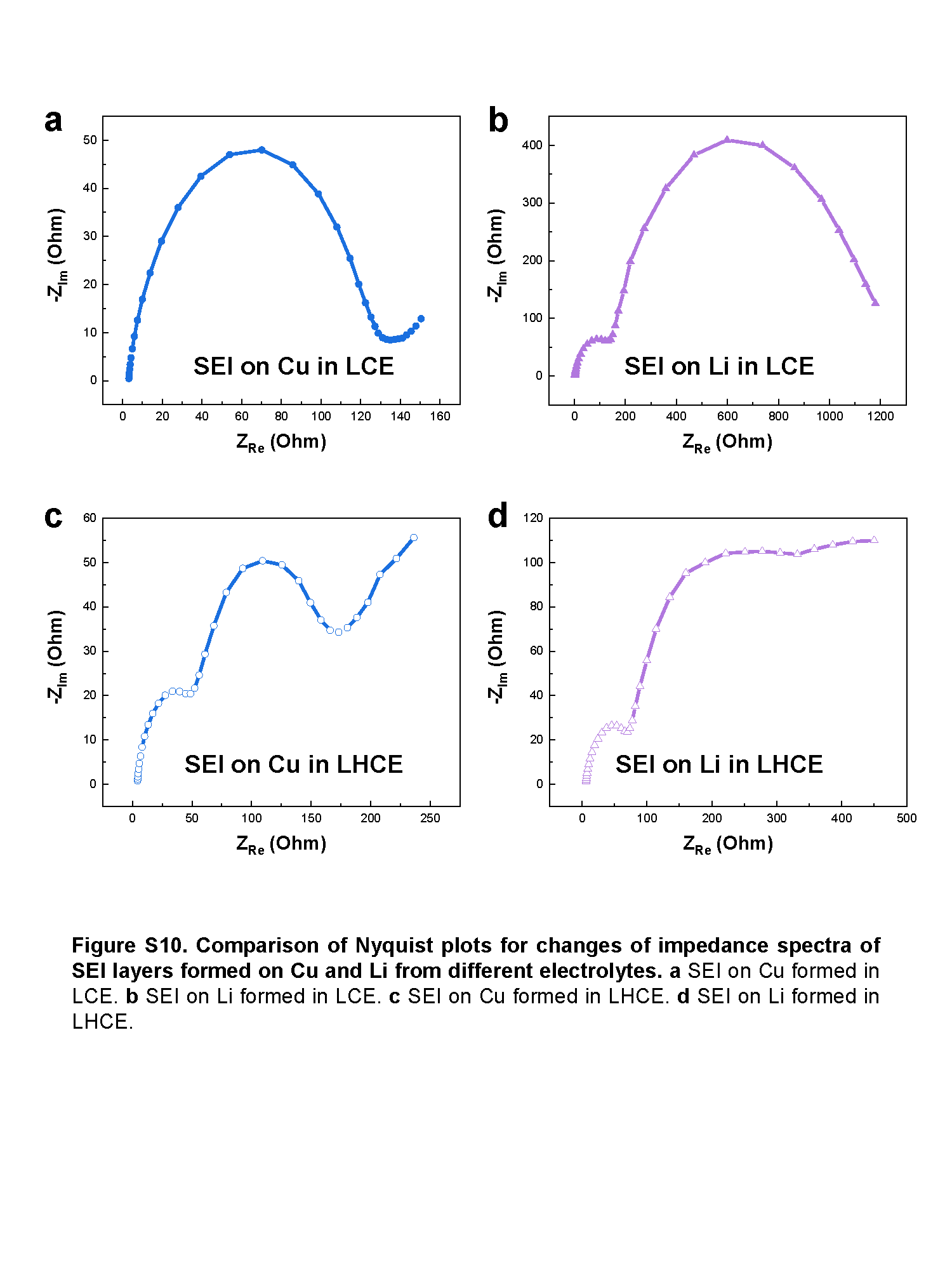
**

**Figure S12. Comparison of Nyquist plots for changes of impedance spectra of SEI layers formed on Cu and Li from different electrolytes. a.** SEI on Cu formed in LCE. **b**. SEI on Li formed in LCE. **c**. SEI on Cu formed in LHCE. **d.** SEI on Li formed in LHCE.


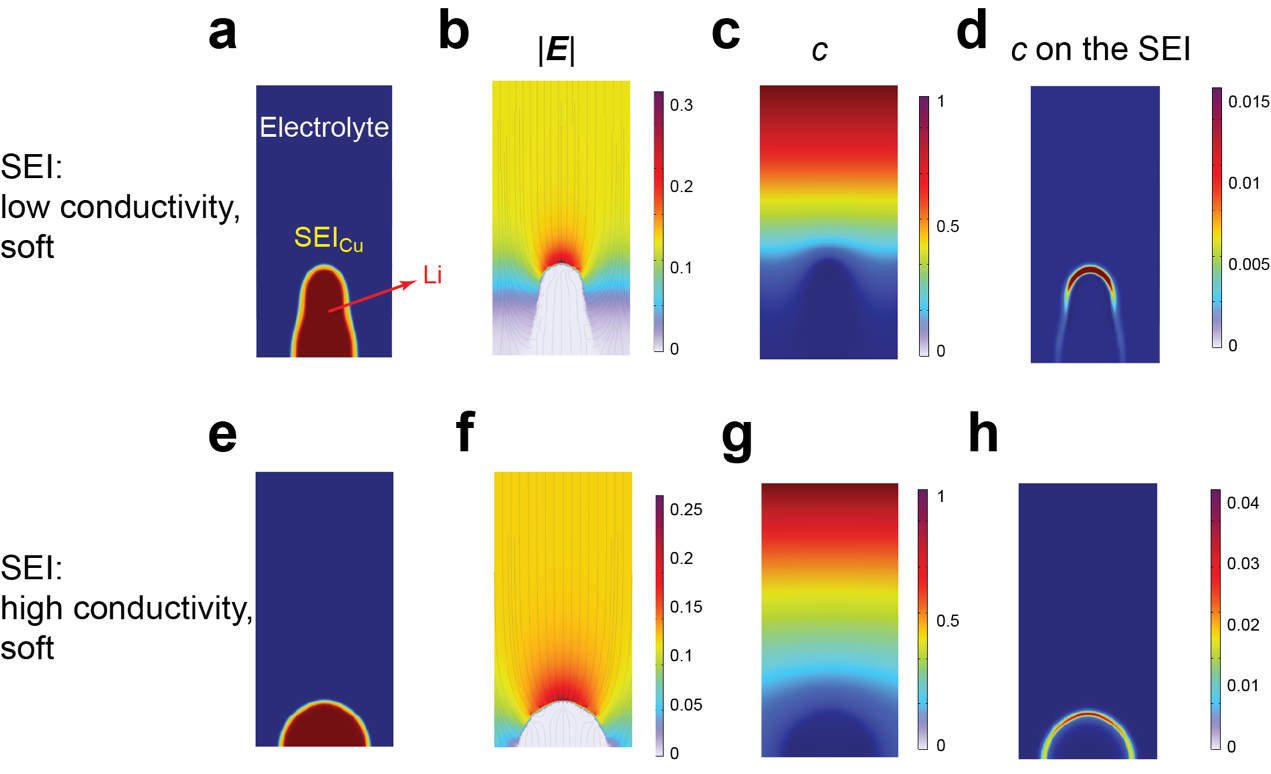


**Figure S13. Phase-field simulations of Li deposition under different SEI ionic conductivities (both mechanically soft)**. Snapshots are taken at the same time for each case. **a, e.** Li growth morphology; **b, f.** Magnitude of the electric field $|\boldsymbol{E}|$, (unit: $N/\mu m$); **c, g.** Li^+^ concentration; and **d, h.** Li^+^ concentration on the SEI. Low ionic conductivity of SEI (**a-d**) results in intensified electric fields and highly concentrated Li^+^ ions at the tip, favoring whisker-like Li growth morphologies, whereas high ionic conductivity of SEI (**e–h**) results in more uniform distribution of Li^+^ ions along the interface, promoting a particle-like Li morphology.


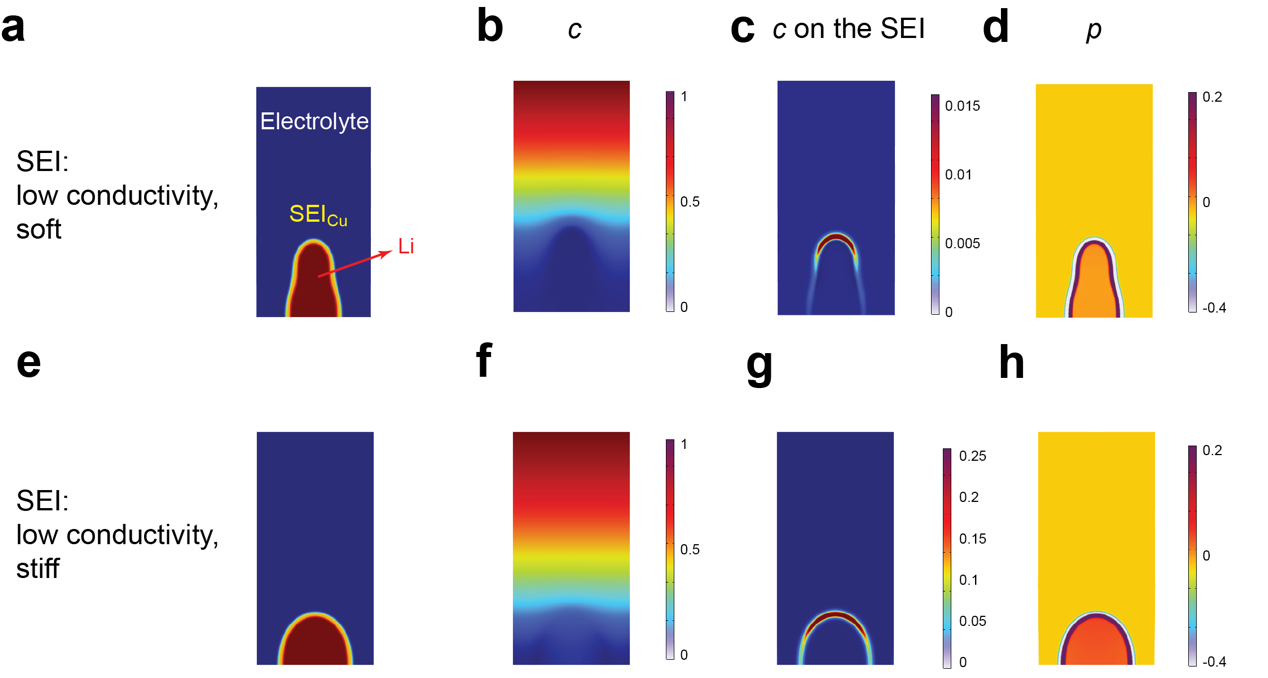


**Figure S14. Phase-field simulations of Li deposition under different SEI stiffness (both low ionic conductivities)**. Snapshots are taken at the same time for each case. **a, e.** Li growth morphology; **b, f.** Li^+^ concentration; **c, g.** Li^+^ concentration on the SEI; and **d, h.** hydrostatic pressure on the SEI (unit: GPa). Under low ionic conductivities, Li^+^ ions in both cases are concentrated at the tip (**c, g**), but stiffer SEI results in higher pressure (**h**) in the Li deposition, inhibiting biased Li deposition and favoring particle-like Li growth morphologies.


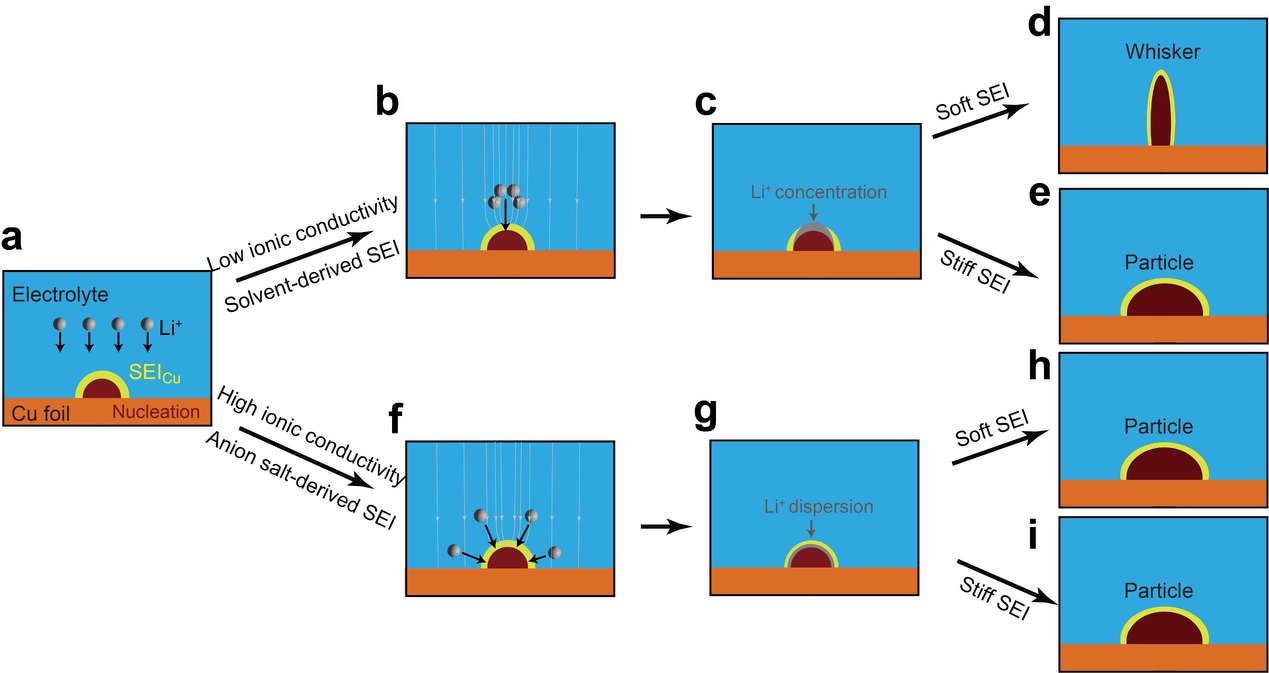


**Figure S15. Schematic of Li growth and SEI evolution. a.** Nucleation within the SEI layer formed on Cu current collect. With low ion conductivity, **b.** Li^+^ ions movement, **c.** Li^+^ ions concentration, and can be divided into dominant SEI layer and minor SEI layer, nucleation and growth of Li within the dominant SEI layer and minor SEI layer with be different**. b.** nucleation of Li at interface between Cu and the dominant SEI. **c.** Growth of Li metal growth morphology **d.** whisker in soft SEI and **e.** particle in stiff SEI. With high ion conductivity, **f.** Li^+^ ions movement, **g.** Li^+^ ions concentration, and Li metal growth morphology in **h.** soft SEI and **i.** stiff SEI.**f**. nucleation of Li at the minor SEI derived from salt anion. **g.** Li particle growth. **h.** Li growth leads to stretching of SEI, which makes SEI formed on Cu thinner. **i.** thin SEI layer growth leads to double structured SEI layer.

**Table S1. Composition information acquired from SEI layers on Li formed in the ether-based LHCE.**

**
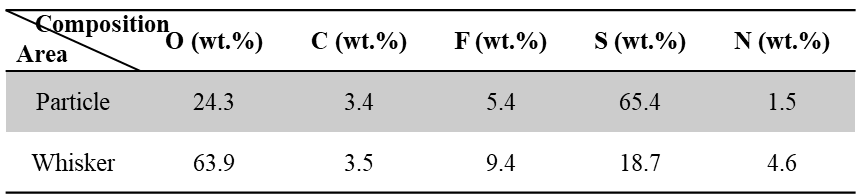
**

**Table S2. Composition information acquired from SEI layers on Li formed in the carbonate-based LCE.**


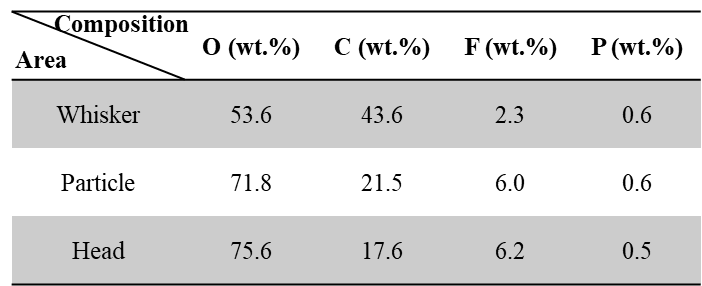


**Table S3. Composition information acquired from SEI layers on Cu formed in the ether-based LHCE.**


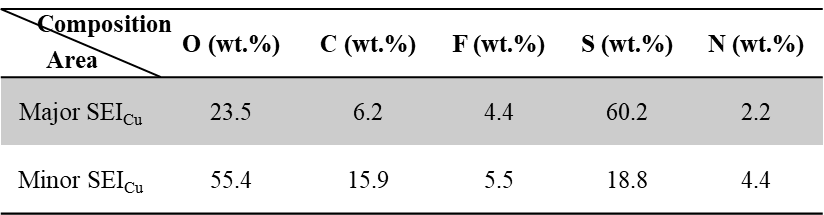


**Table S4. Composition information acquired from SEI layers on Cu formed in the carbonate-based LCE.**


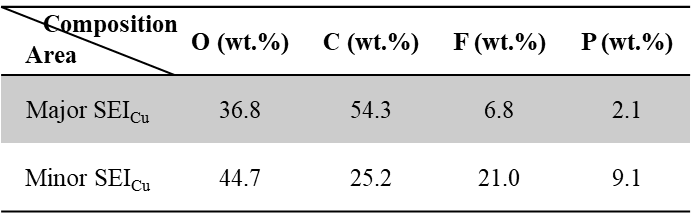


**Table S5. Parameters in phase field simulation.**

|  | Parameter | Symbol | Value | Unit | Source |
| --- | --- | --- | --- | --- | --- |
| Electro-chemistry | Interface mobility | $L_{\xi}$ | $2.5\times{10}^{-6}$ | $m^{3}/(J\cdot s)$ | ^1^ |
|  | Reaction constant | $L_{\eta}$ | $1.0$ | $1/s$ | ^2^ |
|  | Barrier height | $W$ | $1.2\times{10}^{8}$ | $J/m^{3}$ | ^3^ |
|  | Gradient coefficient | $\kappa$ | $3.75\times{10}^{-8}$ | $J/m$ | ^3^ |
|  | Ionic diffusivity | $D_{c}^{\mathrm{LM}}$ | $1\times{10}^{-15}$ | $m^{2}/s$ | ^1^ |
|  | Ionic diffusivity | $D_{c}^{E}$ | $2\times{10}^{-12}$ | $m^{2}/s$ | ^5^ |
|  | Relative permittivity | $\varepsilon_{\phi}^{\mathrm{LM}}$ | $5\times{10}^{4}$ |  | ^1^ |
|  | Relative permittivity | $\varepsilon_{\phi}^{E}$ | $50$ |  | ^1^ |
|  | Symmetry factor | $\alpha$ | 0.5 |  | ^1^ |
|  | Bulk density | $c_{s}$ | $7.64\times{10}^{4}$ | $mol/m^{3}$ | ^1^ |
|  | Bulk density | $c_{0}$ | $4.2\times{10}^{3}$ | $mol/m^{3}$ | this work |
| Mechanics | Young’s modulus | $E_{\mathrm{Li}}$ | $4.9\times{10}^{9}$ | $J/m^{3}$ | ^6^ |
|  | Poisson’s ratio | $v$ | $0.3$ |  | this work |
|  | Eigen-strain | $\varepsilon^{00}$ | 0.01 |  | this work |

**References**

(1) Xue, D.; Fincher, C.; Fang, R.; Sheldon, B. W.; Chen, L.-Q.; Zhang, S. Dynamic interplay of dendrite growth and cracking in lithium metal solid-state batteries. *J. Mech. Phys. Solids* **2025**, *202*, 106197. DOI: <https://doi.org/10.1016/j.jmps.2025.106197>.

(2) Chen, L.; Zhang, H. W.; Liang, L. Y.; Liu, Z.; Qi, Y.; Lu, P.; Chen, J.; Chen, L.-Q. Modulation of dendritic patterns during electrodeposition: A nonlinear phase-field model. *J. Power Sources* **2015**, *300*, 376-385. DOI: 10.1016/j.jpowsour.2015.09.055 (acccessed 2023-06-12T16:46:59).

(3) Tantratian, K.; Yan, H.; Ellwood, K.; Harrison, E. T.; Chen, L. Unraveling the Li penetration mechanism in polycrystalline solid electrolytes. *Adv. Energy Mater.* **2021**, *11* (13), 2003417. DOI: 10.1002/aenm.202003417 (acccessed 2023-06-23T18:23:18).

(4) Bazant, M. Z. Theory of chemical kinetics and charge transfer based on nonequilibrium thermodynamics. *Acc. Chem. Res.* **2013**, *46* (5), 1144-1160. DOI: 10.1021/ar300145c (acccessed 2023-06-12T18:53:06).

(5) Brugge, R. H.; Chater, R. J.; Kilner, J. A.; Aguadero, A. Experimental determination of Li diffusivity in LLZO using isotopic exchange and FIB-SIMS. *J Phys. Energy* **2021**, *3* (3), 034001. DOI: 10.1088/2515-7655/abe2f7.

(6) Masias, A.; Felten, N.; Garcia-Mendez, R.; Wolfenstine, J.; Sakamoto, J. Elastic, plastic, and creep mechanical properties of lithium metal. *J. Mater. Sci.* **2019**, *54* (3), 2585-2600. DOI: 10.1007/s10853-018-2971-3.
